# On the Dependability and Feasibility of Layperson Ratings of Divergent Thinking

**DOI:** 10.3389/fpsyg.2018.01343

**Published:** 2018-08-13

**Authors:** Richard W. Hass, Marisa Rivera, Paul J. Silvia

**Affiliations:** ^1^College of Humanities and Sciences, Thomas Jefferson University, Philadelphia, PA, United States; ^2^Department of Psychology, University of North Carolina at Greensboro, Greensboro, NC, United States

**Keywords:** generalizability theory, consensual assessment technique, divergent thinking, creativity, originality

## Abstract

A new system for subjective rating of responses to divergent thinking tasks was tested using raters recruited from Amazon Mechanical Turk. The rationale for the study was to determine if such raters could provide reliable (aka generalizable) ratings from the perspective of generalizability theory. To promote reliability across the Alternative Uses and Consequence task prompts often used by researchers as measures of Divergent Thinking, two parallel scales were developed to facilitate feasibility and validity of ratings performed by laypeople. Generalizability and dependability studies were conducted separately for two scoring systems: the average-rating system and the snapshot system. Results showed that it is difficult to achieve adequate reliability using the snapshot system, while good reliability can be achieved on both task families using the average-rating system and a specific number of items and raters. Additionally, the construct validity of the average-rating system is generally good, with less validity for certain Consequences items. Recommendations for researchers wishing to adopt the new scales are discussed, along with broader issues of generalizability of subjective creativity ratings.

## 1. Introduction

Creativity is a complex construct, with many different operational definitions (e.g., Plucker et al., 2004). Though not a perfect proxy for creativity, for over 60 years, divergent thinking tasks have commonly served as the operational definition of choice for many creativity researchers. This is becoming increasingly common in the burgeoning neuroscience literature on creativity (e.g., Benedek et al., 2014; Madore et al., 2017; Wang et al., 2017; Beaty et al., 2018; Chen et al., 2018; Vartanian et al., 2018). Though some scoring methods are straightfoward (e.g., fluency), there is considerable disagreement about how to quantify “originality,” “creativity,” or “novelty,” of the responses themselves. Methods for doing so generally fall into two groups: “objective” methods that use frequency-based methods and “subjective” methods that rely on groups of raters to make decisions about different responses. A full discussion of the merits of each method is out of the scope of the current paper, however, this paper builds on a study by Silvia et al. (2008, Study 1) that dealt with such details. The interested reader is urged to read that paper in full, along with several replies (e.g., Kim, 2008; Mumford et al., 2008; Runco, 2008) to gain a fuller perspective on the issues.

This paper took aim at constructing a new subjective scoring system. This new system was developed by considering divergent thinking tests from the perspective of performance assessment. In the words of Shavelson et al. (1993), “a performance assessment [is] a concrete, goal-oriented task …performed by a [person] on a particular occasion …and evaluated by an expert rater who takes into account the process of accomplishing the task as well as the final product” (p. 216). What is important about that definition is that it highlights the importance of considering both process and product, two of the “several” p's that are often cited as different perspectives on assessing creativity (the other two being person and press). The process by which someone arrives at a particular divergent thinking response obviously cannot be totally revealed by only looking at responses, but the scoring system developed for this study was assembled with the process in mind. Also, the process by which someone generates a single divergent thinking response is not the same as the process by which an artist produces a painting. With regard to the latter, the term“process” refers to a collection of cognitions and behaviors that the artist paces through in the course of developing a work. However, from the perspective of performance assessment, it should be feasible to design systems for scoring specific tasks in terms of the goal of the task. That is, if it can be demonstrated that the current system works for simple tasks like generating uses for objects, then additional systems can be developed for scoring more elaborate single-session creations such as collages, solutions to science and math problems, and even creative writing tasks.

It is important to note that the working definition of performance assessment above explicitly uses the term *expert* raters. However, it is not clear what kind of expertise is needed to evaluate the creativity of a response to either an Alternative Uses prompt or a Consequences prompt, or whether training individuals to rate these responses is even necessary. Indeed, divergent thinking tests were designed to be used with children and adults (cf. Wallach and Kogan, 1965; Torrance, 1966), with no special training. As such, we asked whether reliable ratings could be obtained from raters without specialized training. This is both a theoretical and practical concern, as in the former respect, lay people correspond to the “field” of evaluators in the Systems Theory of creativity (Csikszentmihalyi, 1999). In terms of the latter, many researchers do not have access to dedicated research assistants to serve as raters. For the purposes of this study, adults were recruited from Amazon Mechanical Turk, and given instructions, but no special training, on how to apply the rating systems. To the extent that their ratings are reliable and valid, it would mean that with only a small budget, researchers could obtain reliable ratings for divergent thinking responses when research assistants or graduate students are not on hand.

To examine the reliability of the new system, and to consider potential other sources of variation beyond that which classical test theory (CTT) allows, the current study employed generalizability theory (Cronbach et al., 1972). As will be explained generalizability theory (G-theory) is in some sense a generalization of the methods of CTT such that sources of measurement error that are confounded in the CTT system become decoupled in the realm of G-theory (Brennan, 2010). Somewhat confusingly, reliability of measurements in G-theory is usually spoken of in terms of dependability, such that a reliable system of measurement can be depended upon to provide accurate measures for various purposes. Silvia et al. (2008) used this system to examine the dependability of raters, and this study represents an extension of that study to both a new rating system, and a different population of raters.

### 1.1. Tailoring rating scales to task-specific performance

It can be argued that on their faces, the Alternative Uses and Consequences tasks require slightly different approaches to responding, and thus, might need different criteria for scoring. Hass (2017a) showed that much of what likely takes place during generation of Alternative Uses responses is that the prompt object is used as a cue to the memory system to search for either known uses or perceptual and semantic properties of the prompt that would afford some novel use. However, responding to consequences prompts (e.g., “Imagine the consequences of people no longer needing to sleep”), likely requires some form of counterfactual reasoning; the participant must consider a non-existent state of the world and answer a series of self-generated “what-if” questions. The Silvia et al. (2008) system corresponds with the degree to which participants can search for semantically remote associations when responding to Alternative Uses prompts (Hass, 2017b), and scores derived from those ratings have repeatedly been associated with higher fluid intelligence abilities (e.g., Nusbaum and Silvia, 2011; Beaty and Silvia, 2012; Silvia et al., 2013; Kenett et al., 2016) as well as connectivity with the default mode network of the brain (Beaty et al., 2015, 2018). However, Consequences prompts have not featured prominently in cognitive or neuroscience work, which may be a function of their less than adequate reliability in the Silvia analysis. Thus, one obvious benefit to deriving a more dependable system that can be applied to consequences responses is that it will enable researchers to explore similar effects to those described above, using the consequences task as a proxy measure of creative thinking.

As will be described below, the system developed as a part of the current analysis was designed to provide parallel scales for Alternative Uses and Consequences responses that differed with respect to key semantic differentials. Two scales resulted, each with nearly identical semantic anchors, but with slightly different criteria on the higher end of the scales that reflect the differences in what creative thinking entails across the two tasks. Importantly, the goal was not to obtain a scale that would quantify the absolute creativity of the response itself, but rather to quantify the degree to which the response showed evidence of creative thinking on the part of the person generating it. Taken at face value, a high score on such a divergent thinking task should represent the potential to think creatively, and the scale steps were designed with that notion in mind. The concept is similar to existing scales of elaboration (Torrance, 1966) that are used to award points for responses that illustrate more depth of thought.

Table 1 lists the scales as they were given to the raters (instructions given to raters are described in the Method). It should be noted that the word *creativity* does not appear among the descriptors in Table 1. This was purposeful since emerging research on implicit theories of creativity illustrates that people often harbor idiosyncratic conceptions of creativity that might bias their ratings (Hass, 2014; Baas et al., 2015; Hass and Burke, 2016). However, the raters were told that the scales were designed to measure creativity, and that the responses that were generated in creative thinking tasks.

**Table 1:** Statements that formed the rating scale for alternative uses and consequences ratings obtained from MTurk workers.

**Rating**	**Alternative uses**	**Consequences**
1	Very obvious/ordinary use	Very obvious consequence
2	Somewhat obvious use	Somewhat obvious consequence
3	Non-obvious use	Non-obvious consequence
4	Somewhat imaginative use	Somewhat imaginative/detailed consequence
5	Very imaginative/re-contextualized use	Very imaginative/detailed consequence

The scales were formulated by attempting to distill the three criteria suggested by Silvia et al. (2008) into one scale. Obviousness was used instead of commonness for the lower anchors, as it is the opposite of surprising, a quality of creative products that some have argued is often missing from existing scales (Simonton, 2012). A level of shock experienced by the observer or rater has also been suggested to be essential in of determining levels of creativity about products or responses (Bruner, 1962). It is also seen as analogous to commonness as when evaluating commonness, it is plausible that raters would evaluate how obvious the response was, that is, they might evaluate commonness in terms of whether the response might come to mind easily, or easily be reasoned to apply to the task. Another reason to avoid commonness is that raters may be biased by the distribution of responses in the particular dataset they are viewing. That is, they may deem a response to be “common” simply because many people thought of it, not because it represents a relatively uncreative response. For example, it is quite common for participants to describe uses for bricks that involve decorating or making art. Clearly those uses are different than using a brick for building something, and we felt that obviousness captured the difference.

The top anchor for the Alternative Uses task, was designed to be applicable to cognitive studies of creative thinking that stress recombination and re-representation of object properties as key to the divergent thinking process (e.g., Chrysikou et al., 2016; Olteţeanu and Falomir, 2016). For the Consequences task, the top anchor was designed to capture imagination and elaboration, the latter being a scoring system sometimes used for items from the Torrance Tests (Kim, 2006). As will be described, this divergence of the two systems was the motivation for defining task family as a “fixed facet” in the generalizability analysis.

Theoretically, the issues involving inter-scale semantic differences cannot be addressed via reliability analysis, so we also conduct a simple construct validity analysis to examine whether the newly obtained ratings converged with ratings provided by raters from Silvia's original study. Among other reasons, we wouldn't expect perfect agreement with the Silvia et al. (2008) ratings because of the slight shift in scale anchors. Although the scale facets used in the two rating systems are conceptually similar, the prior scoring yielded only holistic rater estimates of creativity, so it is difficult to unpack how different raters weighted or integrated the 3 dimensions of creativity proposed by Wilson et al. (1953) when giving their ratings (cf. Forthmann et al., 2016b). However, if the ratings generated using the new scale correlate at least moderately with the scores derived from the well-established Silvia system, it would be evidence for convergent validity between the two systems, and would stand as preliminary positive evidence for the construct validity of the new system.

#### 1.1.1. Lay people vs. trained raters

Aside from obtaining ratings that might map onto the cognitive processes involved in generating divergent thinking responses, the more practical goal of this study was to probe whether reliable scores could be obtained using raters recruited from sites such as Amazon Mechanical Turk. Much has been written about the reliability and validity of subjective creativity rating systems (e.g., Amabile, 1982, 1983; Kaufman and Baer, 2012). All of these systems are based on the subjective definition of creativity given by Amabile (1982) that something is creative to the extent that people agree that it is (see Amabile, 1982, p. 1001). Perhaps due to the variety of tasks used by creativity researchers, some of which require a great degree of skill (e.g., drawing), it is reasonable to argue that the judges or raters who provide subjective assessments of creativity should have some expertise in the domain (e.g., have studied drawing, know about art history, etc.). However, there is no special expertise required for divergent thinking task performance, with the exception of being adequately literate. In that sense, as long as the raters who evaluate responses to divergent thinking tasks are familiar with the task prompts, they ought to be able to provide valid ratings.

Despite the lack of necessity of expertise for rating divergent thinking responses, many researchers use research assistants to provide creativity ratings, possibly because such people can be trained to use the scoring system in a particular way. For example, some authors pre-categorize responses that seem alike—equating, say, “doorstop” with “prop a door open”—reducing the overall number of subsequent ratings. Such a scheme does require training, and likely some discussion and revision of such a response reduction due to the blurry distinctions between category distinctions. The system presented in this paper does not require this preprocessing step, and the generalizability analysis will examine whether reliable ratings can be obtained by leaving similar responses worded as participants typed or wrote them. To promote this kind of reliability, raters were explicitly instructed to be consistent in awarding their ratings to similar responses. They were also told that they may see many different versions of the same response (they were given the doorstop example), and that they should try to award similar ratings to such similar responses.

Finally, there were two reasons to use MTurk workers as raters rather than research assistants for the current analysis. First, one risk of using trained laboratory assistants as raters is that they may have adopted some implicit bias toward defining creativity as a part of their work with a particular researcher. As such, though there ratings may be dependable, they may not be as valid as those collected by lay people. As a true test of the dependability of this system, we recruited US citizens from Amazon Mechanical Turk, and gave instructions only on how to apply consistent ratings. As mentioned earlier, laypeople vary with regard to their implicit understanding of creativity, so recruiting laypeople for this study allowed for the rater facet of the G-study to encompass such a universe of raters. Second, a practical consideration for some researchers is that research assistants are not always available to provide ratings. Thus, it would be advantageous to pay MTurk workers to do the same job.

### 1.2. A brief overview of generalizability theory

Before describing the details of the study, a brief overview of G-theory is provided here. For more detailed information on theory and computation the reader is referred to the texts by Brennan (2001), Cronbach et al. (1972), and Shavelson and Webb (1991). There are two kinds of analyses that fall under the umbrella of G-theory. First, a *generalizability study* (G-study) is performed in which the error term in CTT is decomposed into various *facets* corresponding to sources of variance. In constructing a G-study, one considers the object of measurement (in this case, a person), and the universe of admissible observations (UAO) of performance by this person. The UAO consists of the levels of the various facets, and in the present case, we considered three facets: raters (r), task family (t), and items (i). This means we are interested in the reliability (or in G-theory, dependability) of a person's score as raters, task families, and items vary. A researcher must also decide whether a facet is considered random or fixed, a decision that affects the scope of the UAO. Random and fixed facets are similar to random and fixed effects in ANOVA. For example, following from (Silvia et al., 2008), raters were considered a random facet in the current study design, meaning that the raters in this study represent a random sample from an infinite universe of raters, all of which are interchangeable. Similarly, the item facet was considered random in the current design, meaning the particular items (e.g., alternative uses for a brick) are also considered as a random sample of a potentially infinite set of prompts for divergent thinking tasks. However, we treated the task family facet as fixed, meaning that our analysis cannot be generalized beyond the two types of items considered: Alternative Uses and Consequences items (tasks). The motivations for the fixed task family facet were two-fold: first, the rating scales used in this study were specifically tailored to these two task families alone. The intent of the study was to advise users of these two task families on how dependable scores should be across different numbers of raters and items. The second motivation was to keep the current design as close to that used by Silvia et al. (2008), while highlighting a hidden facet in that design: the item facet.

The second kind of analysis defined by G-theory is a *decision study* (D-study). As with G-studies, D-studies follow from familiar concepts in CTT, mainly the computation of a reliability coefficient (e.g., coefficient α, Brennan, 2010). To do so, the estimates of variance components computed in the G-study are then used to compute two “reliability-like” coefficients: the generalizability coefficient, 𝔼*ρ*^2^, and the dependability coefficient Φ. The former can be interpreted similarly to coefficient α: it is a ratio of “true-score variance” to “relative-error variance,” essentially “characteriz[ing] error and reliability for decisions based on comparing examinees”(Brennan, 2010, p. 11). The difference between α reliability and the generalizability coefficient, is that relative error variance is calculated based on the partitioned variance components derived from a G-study. In this way, it provides a more accurate metric for measurement error, but the scale of the statistic is the same as α: 𝔼*ρ*^2^ > 0.80 is considered excellent. The dependability coefficient is similar, but is composed of the ratio of true-score variance to “absolute-error variance” and essentially yields a measure of reliability for “absolute (e.g., pass-fail) decisions about examinees” (Brennan, 2010, p. 11). As such, the dependability coefficient is useful for evaluating the “dependability” of any test that is used in that manner, such as an entrance examination. The dependability coefficient will always be less than or equal to the reliability coefficient, due to the differences between relative and absolute error. However, the dependability coefficient can also be interpreted on the same scale as α and the generalizability coefficient (i.e., Φ > 0.80 signifies excellent dependability). As will be noted, the generalizability coefficient applies to most psychological studies, as statistical analysis involves only comparisons of participants. That is, for the current study to show that the current scales are dependable, it suffices illustrate the number of raters and items that yield generalizability coefficients of 0.80 or greater. D-study estimates can be computed for all kinds of designs. For example, we will calculate the generalizability and dependability coefficients for research using 3 raters and two items per task family, and also provide estimates for varying numbers of raters and items. We also provide instructions on how interested researchers can estimate generalizability and dependability coefficients for designs of their choosing.

## 2. Method

### 2.1. Participants

This study used an existing dataset. A random sample of 80 participants were drawn from among the 227 participants who comprised the sample for Study 2 by Silvia et al. (2008). These participants provided responses to two Alternative Uses prompts (brick and knife) and two Consequences prompts (“humans no longer need sleep” and “humans are 12 inches tall”). These data are freely available via Open Science Framework (insert link) along with the analysis scripts and other data files.

### 2.2. Current scoring systems

#### 2.2.1. Average creativity

Ratings for each of 2,091 unique responses across the four prompts were given using the system presented in Table 2. For each participant, an average rating per rater, per response was first constructed for the purposes of generalizability analysis. Following that, an average rating for each prompt was constructed by further averaging the three raters ratings per response, and then calculating the average rating across participants Alternative Uses and Consequences responses separately. Specific instructions provided to the raters are given below.

**Table 2:** Estimated variance components and percent of variance accounted for from the G-study using MTurk average ratings.

**Var source**	**Alternative uses**		**Consequences**	
	**Estimate**	**%**	**Estimate**	**%**
*p*	0.167	24.4	0.067	11.0
*i*	0.069	10.2	0.000	0.00
*r*	0.181	26.6	0.351	56.9
*p* × *i*	0.139	20.4	0.088	14.2
*p* × *r*	0.008	1.2	0.015	2.5
*r* × *i*	0.021	3.1	0.008	1.2
*p* × *i* × *r*, e	0.096	14.1	0.088	14.2

#### 2.2.2. Snapshot scoring

To provide some additional comparison, the snapshot rating system (Silvia et al., 2009) was also used. The snapshot system does not involve ratings of each unique response, but rather requires raters to award a single rating to the set of responses (Runco and Mraz, 1992; Mouchiroud and Lubart, 2001; Forthmann et al., 2016a,b). As such, this required some revision of the instructions and dimensions listed in Table 1 described below. For the purposes of generalizability analysis, there was no need to average scores as each rater assigned one score to each participant for each prompt. For the validity analysis, the three rater scores per participant were averaged per prompt, and those averages were again averaged across the two prompts within each of the Alternative Uses and Consequences tasks.

#### 2.2.3. Fluency

Fluency was also tabulated as the number of responses given to each prompt. For the purposes of validity analysis, an average fluency score was calculated for each of the Alternative Uses and Consequences tasks.

### 2.3. Procedure

#### 2.3.1. Original divergent thinking procedure

Participants recruited by Silvia et al. (2008) were instructed to “write down all of the original and creative uses for a brick” and were further prompted to “write down all of the unusual, creative, and uncommon uses.” Silvia's participants were asked to perform this task within the span of 3 min, repeating the same procedure for the four different scenarios; Alternative uses for a brick, Alternative uses for a knife, creative consequences for if people no longer needed sleep, and creative consequences for if people were 12 inches tall.

#### 2.3.2. Current rating procedure

Three raters (all female, *M* = 47 years old) used the average rating system to rate the creativity of each and every response given by all 80 people. They were asked to complete the ratings over 2 days and were paid $16 each for their work. Raters were supplied with spreadsheets containing the responses and detailed instructions on how to use the scale. The instructions explained that the responses were from participants in a psychology study about creativity, who were asked to generate responses to specific prompts. The prompt (e.g., “Alternative uses for a brick”) was stated as a part of the instructions. They were told that there were no “correct” answers, and reminded to be consistent in applying the ratings, specifically, to assign the same or similar ratings for responses that appeared similar (e.g., “door stop,” “hold a door open” as uses for a brick). Responses to each task prompt were separated into different tabs, and each tab was clearly labeled with the prompt. Participants typed their ratings in the cell next to each response. They were also asked to comment on the ease of use of the scale, and all 3 relayed positive comments, with no rater indicating confusion or lack of ability to rate certain items consistently.

A second set of three raters (2 females, *M* = 32.3 years old) used the snapshot method to rate the set of responses given by each of the 80 participants to each task. These raters were also encouraged to complete the ratings over 2 days and paid $16 for their work. The instructions were identical to the above, except that these raters were told to provide a rating based on the creativity of the majority of each participant's responses. This was done to discourage a correlation between snapshot scores and fluency, such that raters were not simply awarding high snapshot scores to participants who were highly fluent (Forthmann et al., 2016b). Each participant's responses were contained in a single cell in the spreadsheet, with individual responses separated by commas. Raters indicated the rating in the cell adjacent to the list. As with the 3 raters that used the average system, the snapshot raters reported only positive comments about ease of use of the snapshot scale, with none indicating confusion or lack of ability to consistently rate participants.

## 3. Results

All data and analysis scripts are available for download via Open Science Framework (osf.io/bh35a). Data were pre-processed and analyzed within the R Statistical Programming Language (R Core Team, 2016) environment, and using the *gtheory* (Moore, 2016), *psych* (Revelle, 2017), and *lme4* (Bates et al., 2015) packages. Graphics were created using the *ggplot2* (Wickham, 2009) package.

### 3.1. Variable definitions

Since the objects of measurement in this study were persons, each person in the sample (*n* = 80) required a single score from each rater on each item. Four items (two from each family) were administered to participants: two Alternative Uses tasks (brick and knife) and two Consequences tasks. For the purposes of the analyses, the following four scores were calculated for each person in the study for *all four items*: (1) “Silvia average creativity”: an average score per rater per person per item (taken from the Silvia et al., 2008, dataset); (2) “MTurk average creativity”: an average score per person per rater per item using the new system and the MTurk raters; (3) snapshot creativity: a single rating per person, per item from a second set of MTurk raters; and (4) fluency: the number of responses given by each person to each item.

### 3.2. Generalizability study

As previously described, the goal of a G-study to obtain estimates of the decomposed error term described above (i.e., estimates of variance attributable to the object of measurement and the facets) corresponding to a single person, rated by a single rater, on a single item. These estimates are then used as the basis of the D-study, in which generalizability and dependability coefficients can be estimated for designs including varying numbers of raters and items. For each task family (Alternative Uses and Consequences), and for each scoring system, we conducted a *p* × *i* × *r* G-study, which involves estimating 7 variance components: *p*, *i*, *r*, *p* × *i*, *p* × *r*, *r* × *i*, and the confounding of the residual error with the three way *p* × *i* × *r* interaction.

#### 3.2.1. MTurk average scoring

Table 2 provides the results of the G-study for the MTurk average scoring procedure. Several notable results emerged. First, about the same proportion of the variance remains unexplained in both task families (14.1 and 14.2% for the Alternative Uses and Consequences families, respectively). Second, there is nearly twice as much variance attributable to raters on Consequences items (56.9%) than on Alternative Uses items (26.6%). This means that there was more agreement among raters for the average Alternative Uses item than for the average Consequences item. Additionally, the item facet had appreciable variance for the Alternative Uses family (10.2%) compared to no item variance for the Consequences family, meaning that the average amount of creativity did not vary across Consequences items, but did so among the Alternative Uses items. However, both families showed appreciable *p* × *i* variance, such that on average, people's creative performance was not uniform across items within the same family. Finally, though rater variance was high for both items, on average, raters were consistent in applying their ratings across persons, as in both task families, the proportion of variance attributable to the *p* × *r* interaction was low (1.2% for Alternative Uses, 2.5% for Consequences).

#### 3.2.2. Snapshot scoring

Table 3 lists the variance components for the G-study of snapshot scores with task as a fixed facet. There were both differences and similarities between these results and those for the average scores. First, nearly two-thirds of the variability in Alternative uses scores remained unexplained by the facets, and 75% of the variance remained unexplained for the Consequences scores. This is an obvious difference between the two scoring mechanisms, and suggests that a large proportion of variance is attributable either to the three way *p* × *i* × *r* interaction, or some unidentified source of error. Rater variance was relatively large for both task families here, as it was for the average ratings, though the proportions were lower for the snapshot scores (Table 3) due to the high proportion of unexplained variance. There was very little variance left over to be attributed to the other sources and as with the average scores, the patterns of explained variance were different across the two task families.

**Table 3:** Estimated variance components and percent of variance accounted for from the G-Study using snapshot scores.

	**Alternative uses**		**Consequences**	
**Var source**	**Estimate**	**%**	**Estimate**	**%**
*p*	0.098	6.4	0.100	7.1
*i*	0.041	2.7	0.016	1.1
*r*	0.286	18.7	0.161	11.5
*p* × *i*	0.000	0	0.034	2.4
*p* × *r*	0.104	6.8	0.026	1.9
*r* × *i*	0.027	1.8	0.000	0
*p* × *i* × *r*, e	0.971	63.6	1.065	75.9

### 3.3. Dependability study

To facilitate the use of the current data by other researchers, we provide some details on calculating the generalizability coefficient, 𝔼*ρ*^2^, and the dependability coefficient, Φ. The two coefficients are essentially forms of intraclass correlations, and vary according to the definitions of error variance. In the next two sections, we provide generalizabilty and dependability estimates for the current study design (3 raters and 2 items), but also present an overview of the formulas that the reader may use—in consultation with other sources (e.g., Shavelson and Webb, 1991; Brennan, 2001)—to estimate the two coefficients for a design of his or her choosing. Following that, we present estimates of generalizability for a variety of combinations of raters and items.

#### 3.3.1. Generalizability coefficient and relative error variance

The formula for the generalizability coefficient is given below:

(1) $$\begin{array}{l} 𝔼\rho^{2}=\frac{\sigma_{\tau }^{2}}{\sigma_{\tau }^{2}+\sigma_{\delta }^{2}} \end{array}$$

In the equation $\sigma_{\tau }^{2}$ represents *universe score variance*, which is set equal to the variance estimate for persons, and essentially represents true-score variance in CTT. The other term, $\sigma_{\delta }^{2}$ represents *relative error variance*, where relative error is “the difference between a person's observed deviation score and his or her universe deviation score (Brennan, 2000, p. 399).”

The variance in relative errors is estimated using any variance component that involves an *interaction* between person and any random facet. In this study, there are three such terms: the *p* × *i* interaction, the *p* × *r* interaction, and the confounded *p* × *i* × *r, e* residual. Unlike the G-study, these estimates are intended to represent the mean over the number of items and raters in a particular study design, and so each term is divided by the number of different levels of the facet desired for a design. For example, to estimate the relative error variance in a design with 2 items and 3 raters—and thus calculate a generalizability coefficient for that type of study—the variance attributed to the *p* × *i* component would be divided by *n*_*i*_ = 2, the *p* × *r* component would be divided by *n*_*r*_ = 3, and the residual would be divided by the product *n*_*i*_ × *n*_*r*_, or 6. Thus, equation for relative error variance for that design would be:

(2) $$\begin{array}{l} \sigma_{\delta }^{2}=\frac{\sigma_{pi}^{2}}{2}+\frac{\sigma_{pr}^{2}}{3}+\frac{\sigma_{pir,e}^{2}}{6} \end{array}$$

#### 3.3.2. Dependability coefficient and absolute error variance

The dependability coefficient is computed in a similar fashion to the generalizability coefficient, except that relative error variance ($\sigma_{\delta }^{2}$) is replaced with an *absolute error variance* term ($\sigma_{\Delta }^{2}$). In Brennan's (2000) words, “absolute error is the difference between a person's observed score and universe score (p. 342).” The variance of the absolute errors uses all of the estimated variance components in the G-study except for the universe score variance [σ^2^(*p*)], and again each variance component is divided by a number representing the number of conditions in the study for that facet or interaction. As such, absolute error variance involves the same terms as relative error, plus terms for the “main effects” of each facet. Thus, absolute error variance will always be greater than or equal to relative error variance, and so the dependability coefficient will always be less than or equal to the generalizability coefficient. For illustration, absolute error variance for the 2 item, 3 rater design would be:

(3) $$\begin{array}{l} \sigma_{\Delta }^{2}=\frac{\sigma_{i}^{2}}{2}+\frac{\sigma_{r}^{2}}{3}+\frac{\sigma_{pi}^{2}}{2}+\frac{\sigma_{pr}^{2}}{3}+\frac{\sigma_{ir}^{2}}{6}+\frac{\sigma_{pir,e}^{2}}{6} \end{array}$$

The dependability coefficient $\Phi =\frac{\sigma_{p}^{2}}{\sigma_{p}^{2}+\sigma_{\Delta }^{2}}$ is thus a reliability-like coefficient which pertains to the reliability of the scores for “absolute decisions.” An absolute decision might be the decision to include a person in a special creativity program at school based on his or her score on this DT measure. Such decisions are not as common or relevant to psychology researchers, but inclusion of this coefficient in the analysis is informative. However, both coefficients are interpretable on the same scale, with 0.80 or better indicating excellent generalizability or dependability.

#### 3.3.3. D-study estimates for the MTurk average ratings

Table 4 provides the estimated variance components along with the generalizability and dependability coefficients for the current design (3 raters, 2 items). Note that the estimates of universe-score variance [σ^2^(*p*)] are identical to those in Table 2, as D-studies assume that reliability is to be considered for a single person. The Table lists the various *n*′*s* that enter into the formulas above, and each variance component is equal to the variance component from Table 2 divided by the corresponding *n*. As can be seen, the current design did not reach adequate generalizability as 𝔼*ρ*^2^ = 0.65 for the Alternative Uses family, nor for the Consequences family, 𝔼*ρ*^2^ = 0.52. The dependability coefficients are much lower for each family: Φ = 0.47 for Alternative uses and Φ = 0.27 for the Consequences family. These results diverge with those presented by (Silvia et al., 2008), illustrating the importance of including the item facet in the study. Specifically, two items are likely not enough to achieve generalizable or dependable scores with only 3 raters.

**Table 4:** D-study estimates using the MTurk average ratings.

**Estimate**	***n***	**Alternative uses**	**Consequences**
$\sigma_{p}^{2}$	1	0.167	0.068
$\sigma_{r}^{2}$	3	0.060	0.117
$\sigma_{i}^{2}$	2	0.035	0.000
$\sigma_{pr}^{2}$	3	0.003	0.005
$\sigma_{pi}^{2}$	2	0.069	0.044
$\sigma_{ri}^{2}$	6	0.004	0.001
$\sigma_{pri,e}^{2}$	6	0.016	0.015
$\sigma_{\delta }^{2}$		0.088	0.064
$\sigma_{\Delta }^{2}$		0.187	0.182
𝔼*ρ*^2^		0.65	0.52
Φ		0.47	0.27

*Estimates of variance components, relative error variance ($\sigma_{\delta }^{\text{2}}$), absolute error variance ($\sigma_{\Delta }^{\text{2}}$), generalizability coefficient (𝔼ρ^2^), and dependability coefficient (Φ) are for the current design (3 raters, 2 items). Universe-score variance ($\sigma_{\tau }^{\text{2}}$) is $\sigma_{\text{p}}^{\text{2}}$*.

**Figure 1:**
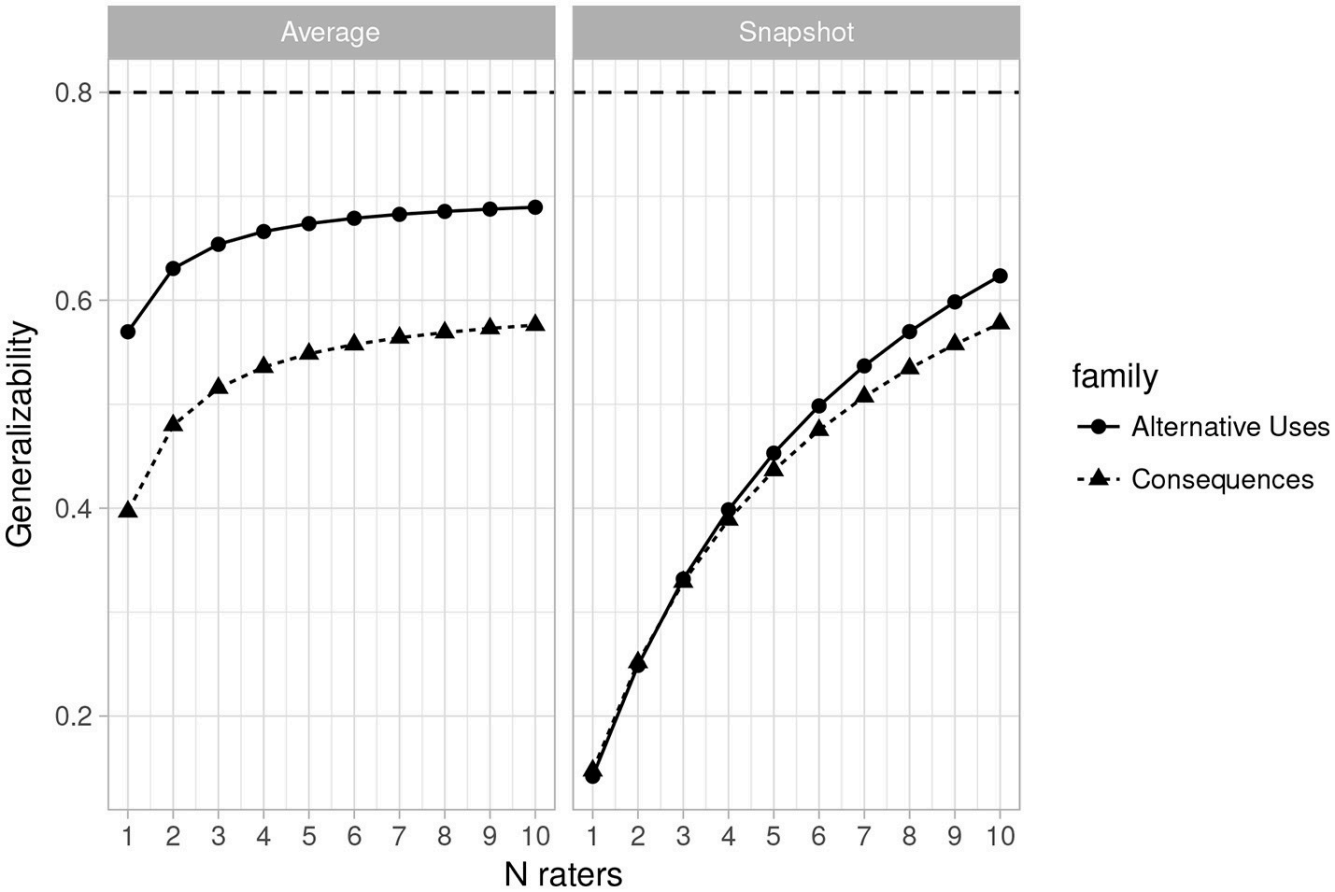
Plot of changes in the generalizability coefficient (𝔼*ρ*^2^) as the number of raters varies from 1 to 10 (*n* items = 2) for Average Ratings **(Left)** and Snapshot Ratings **(Right)**. Horizontal line indicates 𝔼*ρ*^2^ = 0.80.

**Figure 2:**
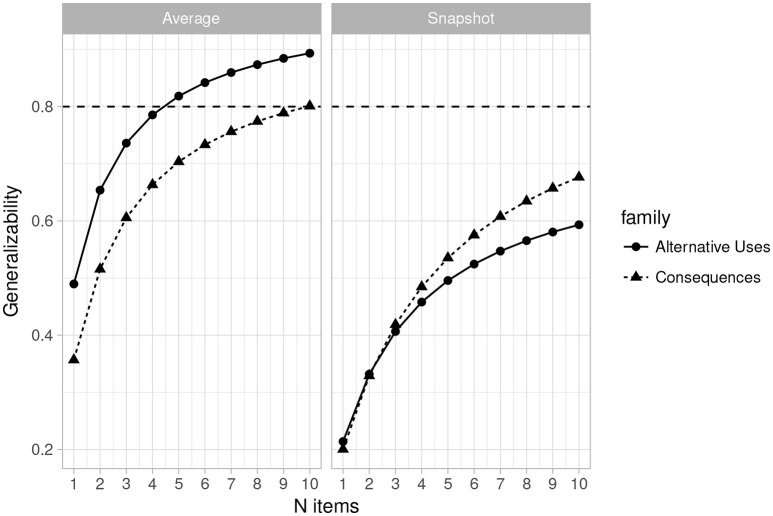
Plot of changes in the generalizability coefficient (𝔼*ρ*^2^) as the number of items varies from 1 to 10 (*n* raters = 3) for Average Ratings **(Left)** and Snapshot Ratings **(Right)**. Horizontal line indicates 𝔼*ρ*^2^ = 0.80.

Figures 1, 2 (left side) give more positive results for the system. Figure 1 is a plot of the generalizability coefficients as the number of raters range from 1 to 10 (fixing n items at 2) and Figure 2 is a similar plot, but with number of items ranging from 1 to 10 (fixing n raters at 3). The horizontal line denotes .80. Figure 1 (right side) shows that designs with 2 items are not optimal for any number of raters between 1 and 10. However, Figure 2 illustrates that with 3 raters, 4 Alternative Uses items would suffice to achieve sufficient generalizability, while at least 8 Consequences items would be required. This is a positive result for the current rating system, though at the cost of having to potentially administer twice as many Consequences items, should that be the choice of task family.

**Figure 3:**
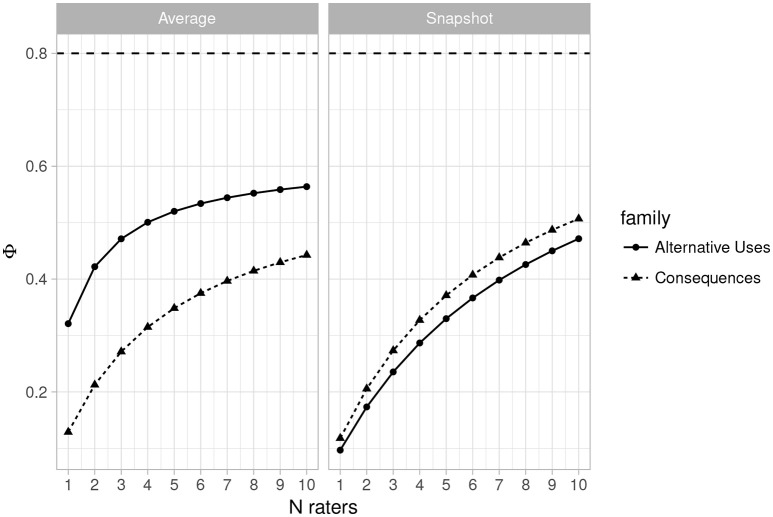
Plot of changes in the dependability coefficient (Φ) as the number of raters varies from 1 to 10 (*n* items = 2) for Average Ratings **(Left)** and Snapshot Ratings **(Right)**. Horizontal line indicates Φ = 0.80.

**Figure 4:**
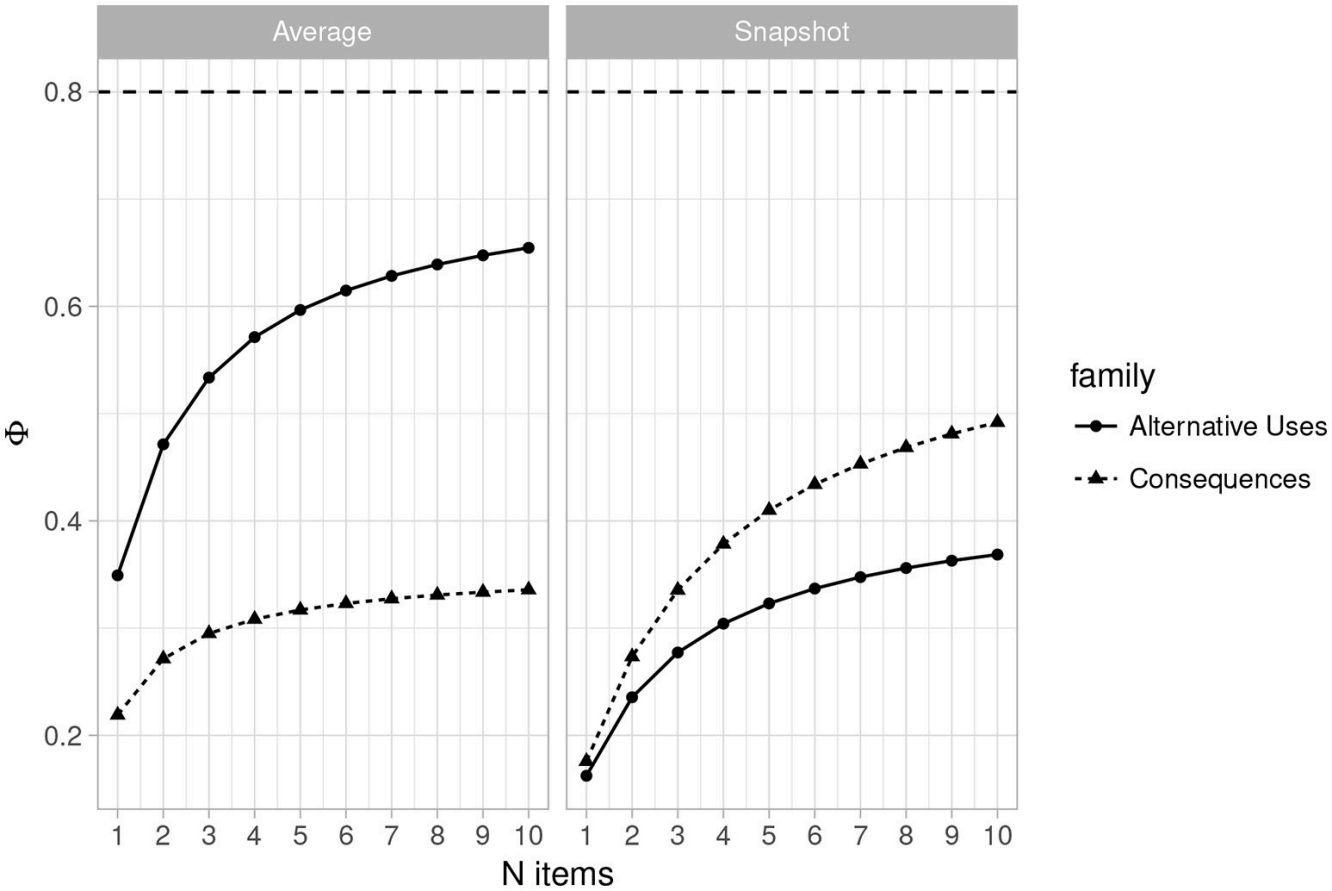
Plot of changes in the dependability coefficient (Φ) as the number of items varies from 1 to 10 (*n* raters = 3) for Average Ratings **(Left)** and Snapshot Ratings **(Right)**. Horizontal line indicates Φ = 0.80.

Figures 3, 4 (left side) illustrate the dependability coefficient estimates over the same ranges as Figures 1, 2. Figures 3, 4 illustrates that dependability remains below 0.80 for all designs and task families. This is not problematic, however, as dependability is less important for most psychological studies, which consist of relative statistical comparisons. That is, generalizability is the more important metric for determining reliability for use in most experimental and individual differences research.

#### 3.3.4. Estimates for the snapshot scores

Table 5 provides the estimated variance components along with the generalizability and dependability coefficients for the current design (3 raters and 2 items) when the snapshot scoring system was used. As with the average rating system, the estimates are quite low, and indeed lower than those obtained for the 3 rater, 2 items design with the MTurk average ratings. Figures 1, 2 (right side) consist of plots of generalizability estimates for the same range of designs described above: fixed items, varying raters (Figure 1), and fixed raters, varying items (Figure 2). Both Figures illustrate the superiority of the average rating system over the snapshot system, and the snapshot system failed to achieve 0.80 for any of the designs considered here. However, the reader can freely estimate how many snapshot items or raters are necessary to achieve adequate reliability. Since the snapshot system involves arguably less work (reviewing 80 sets of responses vs. nearly 2,000 individual responses) a large number of raters and items may be feasible and will likely yield adequate generalizability. Figures 3, 4 similarly illustrate that dependability is quite low for the Snapshot ratings, as it was for the average ratings. Again, since generalizability is more relevant to most psychological studies, this is not seen as problematic. It should be noted, however, that the G-study estimates in Table 3 show that a very large percentage of variance in Snapshot scores is not accounted for by any of the variance components we considered (raters and items). So more research is needed to clarify what other facets may contribute to variability of snapshot scores.

**Table 5:** D-study estimates using the snapshot ratings.

**Estimate**	***n***	**Alternative uses**	**Consequences**
$\sigma_{p}^{2}$	1	0.098	0.100
$\sigma_{r}^{2}$	3	0.095	0.054
$\sigma_{i}^{2}$	2	0.020	0.008
$\sigma_{pr}^{2}$	3	0.035	0.009
$\sigma_{pi}^{2}$	2	0.000	0.017
$\sigma_{ri}^{2}$	6	0.005	0.000
$\sigma_{pri,e}^{2}$	6	0.162	0.117
$\sigma_{\delta }^{2}$		0.196	0.203
$\sigma_{\Delta }^{2}$		0.317	0.265
𝔼*ρ*^2^		0.33	0.33
Φ		0.24	0.27

*Estimates of variance components, relative error variance ($\sigma_{\delta }^{\text{2}}$), absolute error variance ($\sigma_{\Delta }^{\text{2}}$), generalizability coefficient (𝔼ρ^2^), and dependability coefficient (Φ) are for the current design (3 raters, 2 items). Universe-score variance ($\sigma_{\tau }^{\text{2}}$) is $\sigma_{p}^{\text{2}}$*.

### 3.4. Convergent validity

Despite the potential for the MTurk average rating system to achieve adequate reliability, the above results do not address the validity of the ratings. To demonstrate that the current rating scale (Table 1) actually captures the creativity in people's responses, we calculated correlations among all of the scores currently obtained and the scores culled from the raters used by Silvia et al. (2008). Table 6 illustrates that the convergent validity of the new average rating system for alternative uses items with the Silvia ratings is excellent (*r*′*s* > 0.80). Convergent validity is moderate for the new average ratings on the Consequences of being 12 inches tall item (*r* = 0.56), however, the new and old average sleep ratings diverged considerably (*r* = –0.10). This suggests that the sleep item is likely the reason for lower generalizability of the Consequences items rated with the new system, as seen in Figures 1, 2.

**Table 6:** Means, standard deviations, and correlations among fluency, Silvia average scores, MTurk average scores, and snapshot scores.

**Variable**	**M**	**SD**	**Intercorrelations (*****N*** = **80)**														
			**1**	**2**	**3**	**4**	**5**	**6**	**7**	**8**	**9**	**10**	**11**	**12**	**13**	**14**	**15**
1. Silvia Brick	1.69	0.30															
2. Silvia Knife	1.80	0.32	0.52														
3. Silvia 12 Inches	1.41	0.25	0.11	0.14													
4. Silvia Sleep	1.53	0.27	0.17	0.24	0.25												
5. Average Brick (MTurk)	2.32	0.56	0.80	0.51	0.06	0.06											
6. Average Knife (MTurk)	2.72	0.60	0.40	0.82	0.16	0.16	0.50										
7. Average 12 Inches (MTurk)	2.89	0.50	0.25	0.28	0.56	0.32	0.21	0.27									
8. Average Sleep (MTurk)	2.83	0.37	0.20	0.21	0.27	−0.10	0.29	0.27	0.40								
9. Snapshot Brick (MTurk)	2.26	0.70	0.41	0.31	0.17	0.25	0.39	0.19	0.24	0.20							
10. Snapshot Knife (MTurk)	2.59	0.65	0.38	0.57	0.04	0.03	0.40	0.48	0.20	0.33	0.30						
11. Snapshot 12 Inches (MTurk)	2.43	0.80	0.34	0.33	0.30	0.37	0.25	0.26	0.48	0.14	0.34	0.16					
12. Snapshot Sleep(MTurk)	2.64	0.60	0.10	0.19	−0.03	0.27	0.08	0.09	0.10	0.06	0.18	0.22	0.23				
13. Fluency Brick	6.84	2.66	0.12	0.04	−0.07	0.02	0.20	0.09	0.15	0.20	0.26	0.25	0.13	0.18			
14. Fluency Knife	6.36	2.79	0.09	−0.07	−0.10	0.12	0.13	−0.06	0.08	0.08	0.07	0.14	0.08	0.06	0.48		
15. Fluency 12 Inches	6.45	2.70	0.00	−0.02	−0.18	−0.10	0.03	−0.03	0.00	0.02	−0.08	0.04	0.11	0.11	0.31	0.46	
16. Fluency Sleep	6.49	2.71	−0.09	−0.16	−0.04	−0.22	−0.07	−0.20	−0.02	−0.08	−0.01	0.00	−0.04	0.06	0.22	0.25	0.47

*Correlations are Pearson product moment correlations. Fluency scores showed minimal skew. Brick and Knife were the alternative uses items. Twelve inches and sleep were the consequences items*.

Convergent validity of the snapshot system with the original Silvia ratings was moderate, and better for the Alternative Uses items (*r*′*s* > 0.41) than for the Consequences items (*r*′*s* > 0.27). The convergence among Consequences items rated with the two new systems was similar in magnitude as the convergence between snapshot ratings and the Silvia ratings, except for the Consequences of needing sleep item, which again diverged considerably (*r* = 0.06). On a positive note, the bottom rows of Table 6 illustrate the largest fluency-creativity correlation was only (*r* = 0.26), exhibited by the snapshot-brick scores. This illustrates that neither the two new scoring systems—nor the old rating system—are confounded with fluency scores.

Finally, it should be noted that the intra-task-family correlations are moderate for the MTurk average scores, and small to moderate for the snapshot scores. This is also the case for the original ratings obtained by Silvia et al. (2008). Inter-task-family correlations were generally small, which also replicates the data from Silvia et al. (2008). This is a common occurrence in studies using divergent thinking tasks as proxies for creativity, and further illustrates the limits of the UAO for the current G and D studies. This was one of the reasons that task family was modeled as a fixed facet, as it makes clearer the fact that Alternative Uses and Consequences items are not interchangeable with each other across family lines. Rather, it seems that to a certain extent, the items within each task family are somewhat interchangeable.

## 4. Discussion

There were two goals for the current study. First, it was suggested that more specific criteria be used to allow for more equitable reliability across the two task types commonly used by creativity research for assessing idea generation/divergent thinking. Second, it was hoped that the more specific criteria would enable the collection of reliable data when using raters recruited from Amazon's Mechanical Turk system. To a certain extent, those goals were met, especially when MTurkers rated each unique response for all items, and then those scores were averaged per person. However, twice as many raters are necessary to achieve adequate generalizability when Consequences items are administered. On the other hand, the Snapshot system may not yield generalizable or dependable scores even with large number of raters and items. This was due to a large percentage of variance potentially linked to facets not modeled in the current design. So while the Snapshot system may simplify the rating procedure, the scale currently proposed would be more accurately applied by having raters rate each unique response to all items, and then averaging those ratings.

Though the current scales were not designed to perfectly overlap with other systems and scales, the correlations in Table 6 generally suggest that construct validity is adequate for the average rating system when applied to Alternative Uses items, but that there may be less construct validity for the Consequences rating scale. The latter conclusion is unclear, as the ratings applied by Silvia et al. (2008) to Consequences tasks were about as reliable as the current ratings, both of which were less than adequate (cf. Table 4). Despite that, there seemed to be problems with the Consequences scale applied to the “…no longer need sleep” item. Neither the ratings from the average system nor the snapshot system correlated highly with ratings obtained from the original Silvia et al. (2008) study. In addition, there was a low correlation (*r* = 0.18) between average and snapshot scores obtained from the MTurkers for the “sleep” item. Thus, researchers are cautioned that the current rating system used with that item may not yield either reliable or valid creativity scores.

From the perspective of performance assessment, the results of this study are positive. The scale developed for current use was designed to assess creative thinking performance via responses to two types of divergent thinking items. With the task-family fixed in the design, we are limited to recommending the scale for use with only Alternative Uses or Consequences items. However, the convergence of the new ratings with the ratings from Silvia et al. (2008) was strong, especially for Alternative Uses items, meaning that this new system captures creativity, and potentially also captures some aspect of creative thinking performance. The next step for researchers interested in validating the latter statement is to use this system as a performance assessment, potentially testing other psychological hypotheses. Additional G and D studies are necessary to establish whether or not this system can be extended to other task families, or indeed other kinds of creative thinking tasks. However, it should be noted that dependability was lower, so when using these scales to form mean comparisons from different groups of people, a greater number of raters will likely be necessary than when correlational analyses are used, as in individual differences research.

### 4.1. Limitations

Aside from the limits of treating task family as fixed, there are some theoretical and practical limitations that became obvious during analysis. First, It should also be noted that elaboration (detailed responding) was considered as a dimension in the Consequences task, but was not included as a dimension in the Alternative Uses task. So in the latter, people likely rated “doorstop” and “hold a door open” the same, while in the latter, “no need for beds” and “beginning of an energy crisis” would be rated differently. We also did not examine the correlation between the number of words in each response and creativity, which may be important interpreting ratings on the Consequences task. Indeed, recent results illustrate some relation between typing speed and uncommonness of divergent thinking responses culled from online participants (Forthmann et al., 2017). However, the purpose of including detailed/elaborate/imaginative responses on the higher end of the Consequences scale, is that they might represent several steps of reasoning, not that they be necessarily long. That is, in the previous example, “no need for beds” requires one step of logical reasoning (no sleep → no beds), whereas “beginning of energy crisis” requires additional steps (no sleep → up late → darkness → need more light → burn more energy). Further research is necessary to verify that such reasoning is common in Consequences tasks, and may necessitate the revision of the current scale for rating Consequences responses.

Finally, this set of responses was not very large, nor were the number of tasks numerous to potentially fatigue raters. Each rater was able to return his or her ratings within 2 days of receiving the response lists, and no complaints were made via the free response questions raters completed upon submitting their work. However, researchers should be advised that MTurkers are very task-oriented, and need specific instructions on how to perform their tasks, and how to allot their time. That is, it would be advisable to consider whether or not raters should be asked to assess *all* responses, or whether subsets of raters can be assigned specific subsets of responses. This changes the design of our G-study, but such a procedure is easily accommodated within G-theory, though another analysis would be necessary. Similarly, the current set of responses was already “cleaned” of inappropriate or non-sensical responses, and it would be advisable for others to do the same before obtaining ratings, as it is possible that raters would be less reliable when rating such responses.

## 5. Conclusion

The goal of this analysis was to assess whether or not laypeople, recruited from Amazon Mechanical Turk, could provide reliable and valid ratings for divergent thinking responses from the two task families considered. Indeed, we found that using the newly constructed scale reported herein, MTurkers provided reliable and valid ratings, but only when each response in the entire set was rated individually. In addition, the scale was more reliable when used for Alternative Uses items than for Consequences items, and with 3 raters, a study would likely need double the amount of Consequences items to achieve the same level of reliability as a study with only Alternative Uses items. Thus, as a general recommendation, researchers adopting these scales should pay special attention to the choice of items and the choice of raters. For economical designs, 3 raters are generally advisable for Alternative Uses items, and at least 4 items should be used. For studies using Consequences items, researchers can choose between a large number of items and a moderate number of raters, or a moderate number of items and a large number of raters. In either case, the current study provides researchers who lack research assistants with a new method for obtaining creativity ratings for two types of divergent thinking tasks. It is hoped that other researchers adopt and refine these scales, and generate additional scales for more standardization of creativity rating systems across studies.

## Ethics statement

This study was carried out in accordance with the recommendations of the Belmont Report commissioned by the United States Government (1976). The protocol was approved by the University of North Carolina at Greensboro Institutional Review Board. All subjects gave written informed consent in accordance with the Declaration of Helsinki.

## Author contributions

RH and MR conceived of the study and developed the rating scale. PS provided the original dataset. RH performed the data analysis. RH, MR, and PS wrote the manuscript.

### Conflict of interest statement

The authors declare that the research was conducted in the absence of any commercial or financial relationships that could be construed as a potential conflict of interest.

## Supplemental data

All raw and processed data, scripts for analyses and plots are available via the Open Science Framework osf.io/bh35a.
